# U.S. cannabis laws projected to cost generic and brand pharmaceutical firms billions

**DOI:** 10.1371/journal.pone.0272492

**Published:** 2022-08-31

**Authors:** Ziemowit Bednarek, Jacqueline M. Doremus, Sarah S. Stith

**Affiliations:** 1 Orfalea College of Business, California Polytechnic State University, San Luis Obispo, California, United States of America; 2 Department of Economics, University of New Mexico, Albuquerque, New Mexico, United States of America; BeiHang University School of Economics and Management, CHINA

## Abstract

Legalization of cannabis by U.S. states is likely increasing the use of cannabis as an alternative to conventional pharmaceutical drugs. We examined how cannabis legalization between 1996 and 2019 affected stock market returns for listed generic and brand pharmaceutical companies and found that returns were 1.5-2% lower at 10 days after legalization. Returns decreased in response to both medical and recreational legalization, for both generic and brand drugmakers. Investors anticipate a single legalization event to reduce drugmaker annual sales by $3B on average.

## Introduction

Despite federal classification as a Schedule I drug with no medical use and high risk of abuse, by 2020 33 states had legalized medical access to cannabis for severe, debilitating conditions. Yet there is growing awareness of cannabis’ potential therapeutic benefits for a broad range of conditions [1]. By expanding access and, thus use [2], legalization could permit cannabis to compete with conventional pharmaceuticals. Largely unpatentable, cannabis may act like a new generic entrant following medical legalization, leading some individuals to substitute away from other drugs toward cannabis. However, unlike a conventional new generic drug, cannabis use is not restricted to a single or limited set of conditions. This means that cannabis acts as a new entrant across many different drug markets simultaneously. Furthermore, access to recreational cannabis is similar to over-the-counter conventional medications, in that it no longer requires healthcare provider oversight for use.

An emerging body of research examines changes in conventional drug use after state legalization of medical cannabis. Much of this work focuses on subpopulations. For example, legal medical cannabis likely decreases the use of prescription drugs [3, 4], including opioids [5, 6], in Medicaid and Medicare populations. Patient-level studies find legal medical cannabis reduces prescription drug use [7, 8]. Recreational cannabis facilitates use for a broader set of conditions, and may also lead to reductions in prescription drug use if it brings in new patients with unapproved medical conditions or patients who were unwilling or unable to register as medical patients. A study of Medicaid enrollees suggests recreational cannabis legalization reduces the use of lower potency, Schedule III opioids [9], and in a retail setting, legal recreational cannabis decreased over-the-counter sleep aid and antacid sales [10, 11]. Drugmakers appear to recognize the threat and respond strategically, including lobbying against state legalization [12].

Using stock market valuations of publicly traded pharmaceutical firms, we examine how cannabis legalization affects profitability for publicly listed pharmaceutical companies over nearly 25 years of state cannabis legalization. Tools from finance allow us to predict market returns in the absence of legalization using a well-known, empirically validated factor pricing model [13, 14]. Next, we compare realized returns to these predicted returns to estimate how legalization affected drugmaker profits. We contrast investor responses to medical and recreational legalization and for generic and brand drugmakers. From our analysis, we predict how legalization changes conventional drug spending in legal states, including both over-the-counter and prescription medications and all types of patients.

## Study data and methods

We use stock returns and market risk factors to estimate how cannabis legalization affects the market capitalization of drugmakers. In effect, we estimate investors’ expectations of future pharmaceutical firm sales and profitability. We compare drugmakers’ actual and predicted (model-implied) stock returns over the cannabis legalization event window.

### Data

A factor pricing model in finance relates market risk factors to stock returns. Using such a model, we are able to predict theoretical stock returns. For stock returns, we use data on publicly listed companies from the Wharton Research Data Services’ Center for Research in Security Prices (CRSP) and the University of Chicago [15]. Our main outcome variable, the daily stock return, is calculated as the difference between the stock price today and the day before, divided by the price from the day before. For stock prices, we use the adjusted closing stock price from CRSP, which include dividends and stock splits. The daily time series data underlying each of the four factors in the factor pricing model, “Market Excess Return” (MEXT), “Small Minus Big” (SMB, market value), “High Minus Low” (HML, book-to-market ratio), and “Up Minus Down” (UMD, momentum), come from Ken French’s data library, [16]. The variables associated with each factor are created by comparing returns across investment portfolios differentiated by these characteristics. SMB captures differences in returns based on market value of equity (size). In HML portfolios, book-to-market equity ratios are used to compare firms that are “High” or “Low” in terms of the ratio of the value of their book equity to their stock price-based market value. UMD captures differences in historical trends in stock returns that are positive (“Up”) or negative (“Down”). These factors are standard variables used in finance and have been empirically validated as jointly predictive of market returns across a range of markets and time periods, as in [13, 14].

In our main sample, we have 556 companies’ daily stock return data from January 1996 to December 2019. Of these firms, 520 (93%) come from two SIC codes: 2834 (81%) (Pharmaceutical Preparations) and 2830 (12%) (Drugs). Because firms are listed and delisted from the stock exchange over the sample period, the number of distinct firms each year varies from 129 to 266, with the average of 187 and the median of 180 firms. Companies present in the beginning of the sample may have been delisted in the meantime, therefore, the total number (556) is higher than the number of firms for each year separately.

For a subsample of 91 firms, we are able to classify them as brand or generic drugmakers using [17]. In this sample, 16 are brand drugmakers, and 75 are generic drugmakers. Table 1 summarizes total assets, market value, and annual sales for our main sample and our smaller sample of brand and generic drugmakers, in 2019 dollars. In the main sample, the average market value of a firm is $8.9B and average annual sales are $945M. These averages are much higher than the median values and the standard deviations are large, $33B for market value and $2.9B for sales. In the smaller sample of firms, we see that that generic firms tend to be much smaller than brand firms.

**Table 1:** Summary statistics for main and firm type samples.

Type		Mean	Median	SD
Panel A: Main Sample				
	Assets	13539.76	117.72	113523.59
	Market value	8862.44	257.92	32980.23
	Sales	945.15	9.06	2888.11
Panel B: Firm Type Sample				
Generic	Assets	2705.93	126.05	9175.25
	Market value	2493.85	338.92	6630.69
	Sales	265.49	10.91	780.19
Brand	Assets	55433.50	39732.54	49230.92
	Market value	112371.88	95753.76	79440.84
	Sales	7556.70	6683.27	5411.29

Notes: This table presents assets, market value and quarterly sales of companies in our sample. We show data for generic (75 firms), brand (16 firms) and the main sample (556 firms). All amounts are in $MM. We expressed all amounts in terms of 2019 dollars. Among the 91 firms in the Firm Type sample, 4 firms were not in CRSP. For these firms, we manually downloaded historical prices from [17].

**Fig 1:**
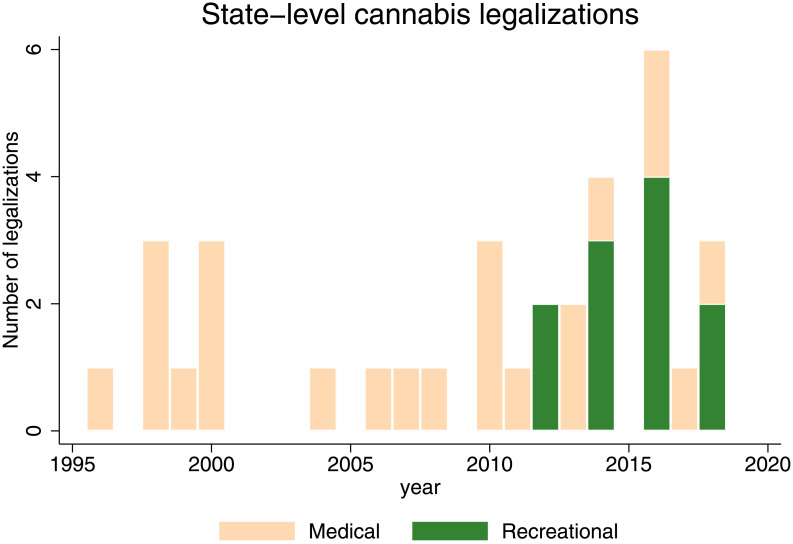
Cannabis legalization events. This figure presents the timing of cannabis legalization events by state.

We paired stock return data with the dates of 45 cannabis legalizations between November 1996 (California) and November 2018 (Oklahoma, Michigan, Mississippi, Virginia and Utah). Each legalization event in the sample is defined as the day that the state governor signed legislation legalizing cannabis or voters approved a ballot initiative to legalize access to cannabis, either medical or recreational. Dates were obtained from two sources: [18, 19]. Fig 1 plots the legalization dates over time and Table 2 reports each state legalization in the sample. We have 34 states (including the District of Columbia) with medical cannabis legalization (15 ballot/19 legislative) and 11 states with recreational cannabis legalization (10 ballot/1 legislative). Among these, we have 12 events with the same legalization dates, and 8 events within 40 days of one another, leading to 28 unique events for analyses of the 40 days surrounding medical and recreational legalization. For each state, medical legalization weakly precedes recreational legalization, and the states which first legalized medical use were also among the first states to pass recreational use laws.

**Table 2:** Medical and recreational cannabis events.

State	Medical	Recreational
California	11/5/1996	11/9/2016
Alaska	11/3/1998	11/4/2014
Oregon	11/3/1998	11/4/2014
Washington	11/3/1998	11/2/2012
Maine	11/2/1999	12/17/2016
Hawaii	6/14/2000	
Colorado	11/7/2000	11/6/2012
Nevada	11/7/2000	11/8/2016
Vermont	5/26/2004	1/22/2018
Rhode Island	1/3/2006	
New Mexico	3/13/2007	
Michigan	11/4/2008	11/6/2018
New Jersey	1/18/2010	
District of Columbia	5/21/2010	11/4/2014
Arizona	11/2/2010	
Delaware	5/13/2011	
Connecticut	5/31/2012	
Massachusetts	11/6/2012	11/8/2016
New Hampshire	7/23/2013	
Illinois	8/1/2013	
Maryland	4/14/2014	
Minnesota	5/29/2014	
New York	7/5/2014	
Montana	11/2/2014	
Pennsylvania	4/17/2016	
Louisiana	5/19/2016	
Ohio	6/8/2016	
Arkansas	11/8/2016	
Florida	11/8/2016	
North Dakota	11/8/2016	
Wisconsin	4/19/2017	
Oklahoma	6/26/2018	
Missouri	11/6/2018	
Utah	11/6/2018	
Count	34	11

Notes: This table presents medical and recreational marijuana legalizations by state, sorted in chronological order. Cannabis legalization dates (enactment) are based on Procon.org, and Powell et al. (2018). Events are measured at the state-level however our analysis is at the national level. Some event occur on the same day in different states. Thus although we have 34 medical events and 11 recreational events, we have have 28 distinct medical dates and 8 distinct recreational dates. When combining both types of events, there are 33 distinct dates.

### Statistical analysis

We use a classic event study framework from finance to first estimate the relationship between a drugmaker’s stock returns and the four standard market risk factors over a period of time well before cannabis legalization. We then use this estimated relationship to predict how drugmaker stock returns would have evolved without cannabis legalization and compare this to realized returns.

#### Estimated relationship between returns and factor prices 150 to 50 days before legalization window

For each drugmaker and each cannabis legalization, we estimate the association between drugmaker stock returns and the four factor variables for 150 to 50 trading days before cannabis legalization using a factor pricing model that adjusts for the four sources of risk associated with the overall stock market exposure: “Market Excess Returns” (MEXT), “Small Minus Big” (SMB, market value), “High Minus Low” (HML, book-to-market ratio), and “Up Minus Down” (UMD, momentum), following [13, 14, 20, 21]. Using data from 150 through 50 trading days prior to the legalization event window, daily firm-level drugmaker stock returns are regressed on an intercept and the four factors in the model, MEXT, SMB, HML and UMD. Our analysis uses the Fama-French-Carhart model. Results are robust to alternative window lengths and baseline models that rely on one risk factor and are more parsimonious [22, 23]. The values of the factor variables are the same for all firms in the analysis, but the intercepts and the coefficients are unique to each firm, and capture the exposure of each firm to the risks captured by the four factor variables. The end result is a set of coefficients and intercepts for 4,689 distinct regressions.

#### Predicting returns around legalization

We use the coefficients from the factor pricing model regressions to predict how returns would have evolved over the 20 days before and after cannabis legalization, had it not occurred. Using data on the four factor returns for this 40-day window, we multiply them by the firm-specific coefficients from the regressions and add these values to the firm’s corresponding intercept to get each firm’s *predicted* returns for the period. These predicted returns are our counter-factual, i.e. they predict what would have happened without cannabis legalization. To relate our framework to the potential outcomes framework [24], we can consider predicted returns as our control group and realized returns as our treated group. Our primary identifying assumptions are that the estimated relationship between drugmaker returns and market factors remains the same between the 100-day period (150-50 days prior to legalization) in which we generated the intercepts and coefficients and the 40-day period in which cannabis legalization occurred, and that investors respond quickly and completely to publicly available information, consistent with the efficient markets hypothesis [25, 26].

We then calculate the difference between the realized (treated) and predicted (control) returns for each day in the 40-day study window, yielding what are called abnormal returns (AR). Finally, we aggregate the abnormal returns over the 40-day study period to estimate daily cumulative abnormal returns (CARs). We compare cumulative returns for realized drugmaker stock prices (treated) and predicted stock prices (control) over the 40-day period surrounding the legalization event to evaluate how drugmaker returns actually evolved as compared to how we would have expected them to evolve without the legalization. More formally, we test whether the daily difference between realized and predicted cumulative returns is statistically significant, calculating t-test statistics for daily CARs, ARs, and differences in ARs across medical and recreational events and generic and brand drugmakers, assuming cross-sectional independence as in [27]. Following the finance literature, our results are robust to the dependence assumption test of [28], and we adjust the standardized residuals for event-induced changes in volatility as in [29] and cross-correlation as in [30].

### Investor response timing

In classic finance studies, most of the public information disseminates quickly with investors responding to events, such as earnings releases, within a short time frame around the event date. In our setting, the timing and magnitude of information dissemination is less clear-cut. Election uncertainty is typically resolved in the two weeks preceding the election [31], with prediction markets operating with only a 1.5 percentage point margin of error in the week prior to major elections [32]. Hence, we anticipate that some of the stock market response will precede the actual date of enactment.

The method of legalization may further affect how early investors respond. Governors often state their intention to sign or veto when the bill passes the legislature, but before the bill reaches their desk, which determines the date of the enactment. In comparison, for ballot initiatives investors have less information about the likelihood that the initiative passes before the formal vote is tallied. Because we expect investors to respond to changes in information and not formal policy dates, and given most recreational cannabis legalization occurred via ballot initiative, we anticipate the stock market response for recreational legalization will begin closer to the date of the enactment (day zero) than for medical legalization.

We expect that the stock market response will persist throughout our event study window, however, the literature indicates that some reversion will occur due to market overreaction that subsequently reverts [33] or responses to noisy information that are later adjusted [34]. Markets which receive less investor attention, e.g., smaller markets, tend to exhibit a more persistent response [35], suggesting that the effect may be more persistent for medical legalization than for recreational legalization and for smaller generic drugmakers than for larger brand ones.

### Estimating changes in drugmaker sales

Using tools from finance, we translate changes in stock returns into sales revenues, with major implications for U.S. spending on conventional pharmaceutical medications. We use a technique applied widely in corporate finance, e.g. [36], to estimate the implied change in sales per share given changes in stock prices using industry-level price-to-sales ratios. These ratios enable one to relate market value (stock price per share times the number of shares) to total sales revenues (sales per share times the number of shares). Estimating the effect of a change in market value on future sales was first established in the seminal work of [37]. For each event, we use the price-to-sales ratio for that year, with the exception of 1996, where we use the value for 1998. The average ratio of price-to-sales for the pharmaceutical industry between 1998 to 2019 was 4.91, with values ranging from 2.57 in 2008 to 8.28 in 1998, based on publicly available data [38]. We start by taking each company’s market value at the beginning of the event. This is estimated as the product of the current price per share and the number of the shares outstanding and is a forward-looking measure of the expected company’s cash flows and their risk. Next, we multiply the market value by the cumulative abnormal return at the end of the event window to obtain the change in each firm’s market value from each event. Lastly, we divide the change in the firm’s market value by the stock price-to-sales ratio to get the change in firm’s annual sales from the event. The total of these estimated changes across all firms listed as of the time of the event yields the annual sales change per event as expected by stock market participants.

### Sensitivity analysis

Three states (California, Massachusetts, and Nevada) all legalized recreational cannabis in November 2016, which also coincided with the election of Donald Trump for president. Because this election rallied pharmaceutical markets [39], we conduct a sensitivity check on our analyses by excluding November 2016 events.

### Limitations

This study has four primary limitations. First, we only observe effects on publicly listed pharmaceutical firms. Although most of the large pharmaceutical manufacturers are publicly listed, some are not, including most notably the opioid manufacturer Purdue Pharma. Because we fail to observe effects for private firms, our results underestimate the effect of legal cannabis on conventional drug spending.

Second, our treatment—cannabis legalization—brings challenges for estimation. As discussed earlier, investors respond to information, not policy events. Our legalization events vary in both the timing of information dissemination and the size of the new legal cannabis market. This heterogeneity will add measurement error, biasing our estimates toward zero. For legalization via ballot initiatives, there is a concern that there may be other ballot measures or electoral outcomes that affect the pharmaceutical industry. If other measures are systematically likely to be listed with cannabis legalization, our measure of legalization’s effect will include the effect from these measures, with an ambiguous net effect on our estimate. This is a greater risk for recreational legalization, which is primarily passed via ballot initiatives.

Third, the event study framework relies on a baseline model that uses historical data to predict hypothetical returns in the absence of the event. We use the canonical asset pricing model in finance, so limitations may come from the selected period for the baseline model (150-50 days before legalization) and the event window (20 days before and after legalization). Our results are robust to varying these window lengths. Poor choices for these windows can reduce the precision of the predicted returns, reducing the statistical power to detect an effect. We followed norms within finance literature for the length and timing of these windows.

Finally, we assume that investors behave rationally. This assumption does not have an impact on the methodology, but rather on the interpretation of the results. However, if a substantial enough fraction of investors act for speculative reasons unrelated to the predicted effect of cannabis legalization on drugmaker sales, we may over- or underestimate the effect of cannabis legalization on drugmaker market value and the implied effect on drug spending in the U.S.

## Study results

### Changes for combined, medical and recreational legalization

**Fig 2:**
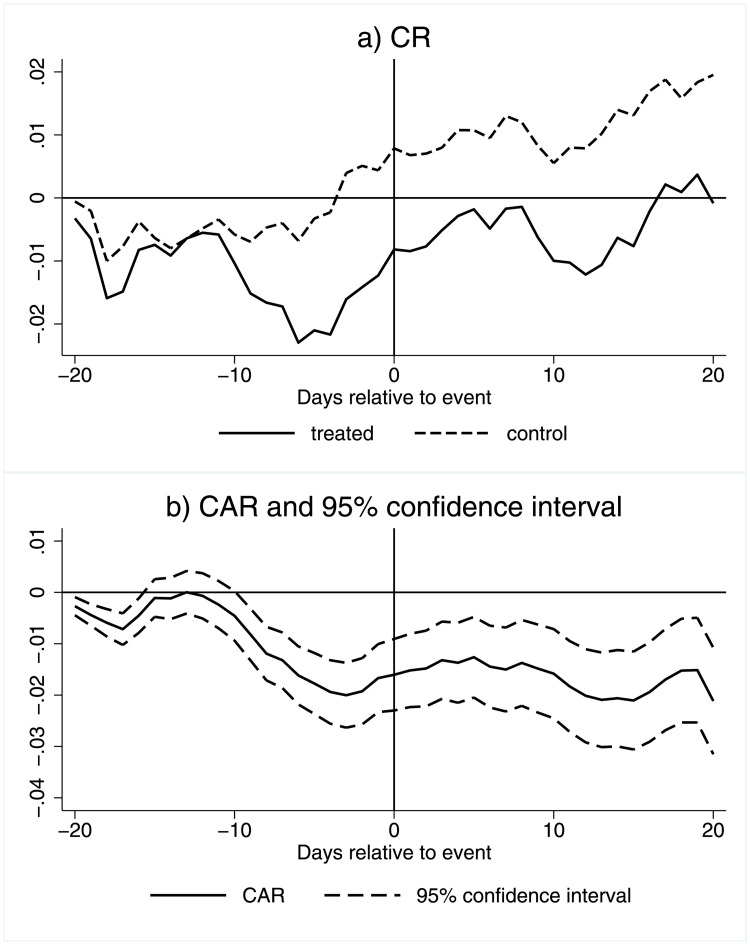
CRs and CARs for drug makers. This figure shows the comparison of cumulative returns for all drug makers. Subfigure a) presents the actual observed cumulative returns (treated) and counterfactual (control, model-implied) cumulative returns around the event window. Subfigure b) presents the cumulative abnormal returns (CAR). CAR is defined as the difference between the treated and control returns. Dashed lines indicate the 95% confidence bands. X-axis is business days relative to legalization event.

Comparing predicted and actual cumulative returns (CRs) for drugmakers reveals changes in investor behavior around cannabis legalization. In Fig 2, the solid line of the top panel shows realized cumulative returns for drugmakers (treated) over time. The dashed line shows predicted returns (control) over time. Table 3 presents t-tests and their precision. We note that the treated and control cumulative returns in Panel (a) are nearly identical until about ten business days before legalization, at which point the treated line steeply decreases while the control line stays about the same. About five days before the event, the two lines become parallel again, though now with a gap of about 1-2% between them. After this point, drugmaker returns once again reflect patterns in the overall market. The estimated cumulative abnormal returns (CARs), i.e. the cumulative difference between treated and control abnormal returns over the event window, and the corresponding 95% confidence interval, are plotted in Panel (b) of Fig 2. The difference is about 1.5-2%, statistically significant, and persists during the 20 business days following the event. This corresponds to a loss of about $133-177M per firm. This is calculated by multiplying the cumulative abnormal returns value, 1.5-2%, by the average market value, $8.86B.

**Table 3:** Abnormal returns (ARs) and cumulative abnormal returns (CARs) around cannabis legislation events.

	All years		No November 2016	
Day	AR	CAR	AR	CAR
-10	-0.0021***	-0.0071***	-0.0023***	-0.0010*
-9	-0.0036***	-0.0110***	-0.0037***	-0.0051***
-8	-0.0041***	-0.0156***	-0.0037***	-0.0093***
-7	-0.0015***	-0.0185***	-0.0004***	-0.0110***
-6	-0.0030***	-0.0220***	-0.0031***	-0.0137***
-5	-0.0021***	-0.0247***	-0.0022***	-0.0162***
-4	-0.0021***	-0.0282***	-0.0009***	-0.0189***
-3	-0.0009***	-0.0303***	0.0002	-0.0194***
-2	0.0011***	-0.0299***	0.0007***	-0.0180***
-1	0.0031***	-0.0261***	0.0032***	-0.0144***
0	0.0005***	-0.0240***	-0.0005***	-0.0134***
1	0.0014***	-0.0229***	-0.0017***	-0.0153***
2	0.0017***	-0.0199***	-0.0007***	-0.0166***
3	0.0020***	-0.0155***	0.0012***	-0.0149***
4	-0.0003*	-0.0148***	-0.0006***	-0.0152***
5	0.0007***	-0.0138***	0.0009***	-0.0142***
6	-0.0021***	-0.0166***	-0.0024***	-0.0173***
7	-0.0003*	-0.0180***	-0.0011***	-0.0194***
8	0.0016***	-0.0164***	0.0003**	-0.0196***
9	-0.0002	-0.0158***	-0.0015***	-0.0208***
10	-0.0009***	-0.0150***	-0.0004**	-0.0210***
N	202276	202317	183211	183252

Notes: We show the combined effect of medical and recreational use legalizations. CARs are calculated using the Fama-French-Carhart model with a 100-day estimation window and a 50-day gap. We calculate ARs and CARs of a buy-and-hold strategy starting 20 trading days before the event. We show mean cross-sectional ARs and CARs (in %) in a 10-day window around the event. Significance of the difference between medical and recreational returns is based on t-test statistics calculated assuming cross-sectional independence as in [27]. Standardized residuals are adjusted for event-induced changes in volatility as in [29], and for event-induced changes in volatility and cross-correlation as in [30].

* indicates significance at 10% level,

** at 5% level, and

*** at 1% level.

**Fig 3:**
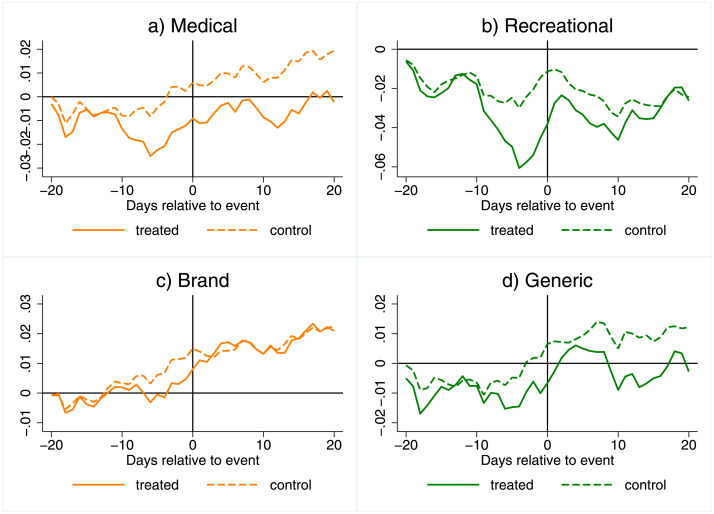
Cumulative returns, by medical/recreational and brand/generic. This figure shows CRs of drug makers. Subfigures (a) and (b) are for medical and recreational events respectively, based on the full sample of 556 companies. Subfigures (c) and (d) show CRs for brand and generic drug makers. Dashed lines indicate the 95% confidence bands.

When we break out investor responses across medical and recreational legalization, we find that much of the average effect in Fig 2 is driven by medical legalization events. In Fig 3, Panel (a) shows CRs for medical legalization and (b) shows CRs for recreational legislation. The results for medical legalizations in Panel (a) look very similar to our results in Panel (a) of Fig 2 in terms of magnitude and the timing of changes between treated and control CRs.

In contrast, the CRs for recreational legalizations in Panel (b) of Fig 3 show three key differences from our overall results: there is a much steeper decrease in the two weeks prior to the election and the difference in cumulative returns between treated and control returns resolves slightly closer to the time of legalization and does not persist. Given fewer recreational legalization events, we have less statistical power and results for recreational markets may be more sensitive to outliers, in particular November 2016. When we exclude November 2016 events, the gap between treated and control CRs is persistent and the departure less steep These results are in Panel (b) of Table 4. The outsized effect of November 2016 on recreational market estimates may be driven by two factors. The steep initial decrease may reflect that California, Nevada, and Massachusetts all legalized recreational cannabis use that year, with California the largest state in the U.S. by population. The lack of a persistent effect when including November 2016 may reflect the unexpected outcome of the U.S. presidential election, which rallied pharmaceutical markets [39].

**Table 4:** Abnormal returns (ARs) and cumulative abnormal returns (CARs) around cannabis legislation events.

**Panel A: ARs for medical and recreational legalizations**						
	All years			No November 2016		
Day	Med.	Rec.	Diff.	Med.	Rec.	Diff.
-10	-0.0027***	-0.0002	-0.0025***	-0.0028***	0.0002	-0.0029***
-9	-0.0034***	-0.0041***	0.0007**	-0.0035***	-0.0047***	0.0012***
-8	-0.0039***	-0.0045***	0.0006*	-0.0039***	-0.0026***	-0.0013***
-7	-0.0014***	-0.0019***	0.0005*	-0.0008***	0.0012***	-0.0020***
-6	-0.0024***	-0.0051***	0.0027***	-0.0023***	-0.0068***	0.0045***
-5	-0.0011***	-0.0057***	0.0045***	-0.0013***	-0.0066***	0.0052***
-4	-0.0011***	-0.0056***	0.0045***	-0.0007***	-0.0019***	0.0012***
-3	-0.0004***	-0.0027***	0.0023***	0.0004***	-0.0010***	0.0014***
-2	0.0015***	-0.0002	0.0016***	0.0013***	-0.0020***	0.0032***
-1	0.0033***	0.0025***	0.0007**	0.0033***	0.0030***	0.0003
0	-0.0006***	0.0043***	-0.0049***	-0.0006***	0.0001	-0.0007
1	-0.0009***	0.0095***	-0.0104***	-0.0023***	0.0011***	-0.0034***
2	0.0006***	0.0055***	-0.0049***	-0.0007***	-0.0008*	0.0002
3	0.0016***	0.0031***	-0.0015***	0.0012***	0.0012***	-0.0000
4	0.0001	-0.0018***	0.0019***	-0.0001	-0.0030***	0.0029***
5	0.0013***	-0.0015***	0.0028***	0.0014***	-0.0014***	0.0028***
6	-0.0020***	-0.0025***	0.0005*	-0.0020***	-0.0044***	0.0024***
7	-0.0001	-0.0010***	0.0010**	-0.0004*	-0.0047***	0.0043***
8	0.0008***	0.0043***	-0.0035***	0.0004***	-0.0003	0.0008**
9	-0.0006***	0.0012***	-0.0018***	-0.0016***	-0.0012***	-0.0003
10	-0.0006***	-0.0018***	0.0012***	-0.0003*	-0.0008*	0.0005
N	157370	44906	202276	151015	32196	183211
**Panel B: CARs for medical and recreational legalizations**						
	All years			No November 2016		
Day	Medical	Recreational	Diff.	Medical	Recreational	Diff.
-10	-0.0068***	-0.0080***	0.0012	-0.0039***	0.0123***	-0.0161***
-9	-0.0105***	-0.0129***	0.0024*	-0.0076***	0.0067***	-0.0143***
-8	-0.0148***	-0.0186***	0.0038***	-0.0118***	0.0026	-0.0145***
-7	-0.0170***	-0.0236***	0.0066***	-0.0134***	0.0003	-0.0136***
-6	-0.0198***	-0.0300***	0.0102***	-0.0154***	-0.0055***	-0.0099***
-5	-0.0213***	-0.0370***	0.0157***	-0.0169***	-0.0130***	-0.0039**
-4	-0.0232***	-0.0457***	0.0225***	-0.0187***	-0.0200***	0.0013
-3	-0.0244***	-0.0511***	0.0267***	-0.0187***	-0.0227***	0.0040**
-2	-0.0234***	-0.0528***	0.0294***	-0.0170***	-0.0228***	0.0058***
-1	-0.0196***	-0.0485***	0.0289***	-0.0135***	-0.0187***	0.0052**
0	-0.0193***	-0.0408***	0.0215***	-0.0132***	-0.0143***	0.0011
1	-0.0203***	-0.0318***	0.0115***	-0.0156***	-0.0139***	-0.0017
2	-0.0189***	-0.0232***	0.0043**	-0.0167***	-0.0163***	-0.0004
3	-0.0157***	-0.0146***	-0.0012	-0.0152***	-0.0135***	-0.0017
4	-0.0150***	-0.0141***	-0.0009	-0.0150***	-0.0158***	0.0007
5	-0.0135***	-0.0151***	0.0016	-0.0136***	-0.0172***	0.0035
6	-0.0159***	-0.0190***	0.0030	-0.0160***	-0.0232***	0.0072***
7	-0.0168***	-0.0222***	0.0054**	-0.0171***	-0.0304***	0.0133***
8	-0.0160***	-0.0179***	0.0019	-0.0169***	-0.0321***	0.0152***
9	-0.0160***	-0.0148***	-0.0012	-0.0183***	-0.0326***	0.0142***
10	-0.0156***	-0.0128***	-0.0027	-0.0185***	-0.0326***	0.0141***
N	157411	44906	202317	151056	32196	183252

Notes: We show separately the effect of medical and recreational use legalizations. CARs are calculated using the Fama-French-Carhart model with a 100-day estimation window and a 50-day gap. We calculate ARs and CARs of a buy-and-hold strategy starting 20 trading days before the event. We show mean cross-sectional ARs and CARs (in %) in a 10-day window around the event. Significance of the difference between medical and recreational returns is based on t-test statistics calculated assuming cross-sectional independence as in [27]. Standardized residuals are adjusted for event-induced changes in volatility as in [29], and for event-induced changes in volatility and cross-correlation as in [30].

* indicates significance at 10% level,

** at 5% level, and

*** at 1% level.

### Changes for brand and generic drugmakers

Next, we use our smaller sample to break out investor responses for generic and brand drugmakers in Panels (c) and (d) of Fig 3 and find that generic drugmaker returns, in percentage terms, likely drive our main result. T-tests and their statistical significance are presented in Table 5. The investor response for generic drugmakers is larger in magnitude and is persistent. In contrast, for brand drugmakers, treated returns depart later from the control, the difference is smaller, and it disappears a few days after the event. The difference in the response across generic and brand drugmakers is statistically significant and persistent. This is clear in columns 3 and 6 of Table 5. A negative number indicates that the generic makers’ response is lower (more negative) than the brand makers’ response. These differences are negative in most cases and become highly statistically significant a few days before the event. The effect persists until the end of the event window. Although generic manufacturers have a greater percentage decrease at the time of enactment, about 1.3% versus 0.7%, the vast difference in market value of generic and brand firms shown in Table 1 means that the decrease in market value for generic firms is much smaller: $32M for generic versus $786M for brand firms.

**Table 5:** Abnormal returns (ARs) and cumulative abnormal returns (CARs) around cannabis legislation events.

**Panel A: ARs for all legalizations, by brand status**						
	All years			No November 2016		
Day	Generic	Brand	Diff.	Generic	Brand	Diff.
-10	0.0011***	0.0005**	0.0007	0.0011***	0.0005**	0.0006
-9	-0.0017***	-0.0006***	-0.0011**	-0.0019***	-0.0009***	-0.0010*
-8	-0.0008**	-0.0009***	0.0001	-0.0010**	-0.0006***	-0.0004
-7	-0.0009***	-0.0029***	0.0020***	-0.0004	-0.0022***	0.0018***
-6	-0.0035***	-0.0005***	-0.0030***	-0.0045***	-0.0005***	-0.0040***
-5	-0.0020***	-0.0003*	-0.0017***	-0.0038***	-0.0006***	-0.0032***
-4	0.0003	-0.0016***	0.0020***	0.0012***	-0.0018***	0.0031***
-3	-0.0005	0.0003*	-0.0007*	0.0002	0.0001	0.0001
-2	0.0021***	-0.0004**	0.0025***	0.0014***	-0.0009***	0.0022***
-1	-0.0040***	0.0011***	-0.0051***	-0.0046***	0.0010***	-0.0055***
0	-0.0011***	0.0002	-0.0014***	-0.0041***	-0.0023***	-0.0018***
1	0.0031***	0.0041***	-0.0010**	-0.0023***	0.0008***	-0.0031***
2	0.0047***	0.0009***	0.0038***	0.0022***	-0.0003*	0.0025***
3	0.0029***	0.0033***	-0.0004	0.0017***	0.0031***	-0.0015***
4	0.0003	0.0012***	-0.0009*	-0.0007	0.0011***	-0.0018***
5	-0.0022***	0.0004**	-0.0027***	-0.0016***	0.0012***	-0.0028***
6	-0.0027***	-0.0018***	-0.0009*	-0.0028***	-0.0012***	-0.0017***
7	-0.0031***	-0.0009***	-0.0022***	-0.0041***	-0.0005**	-0.0036***
8	0.0005*	-0.0005***	0.0011***	0.0009***	-0.0002	0.0011**
9	-0.0021***	0.0002	-0.0023***	-0.0017***	0.0007***	-0.0024***
10	-0.0022***	0.0001	-0.0022***	-0.0031***	0.0005***	-0.0036***
N	27305	15211	42516	24025	13899	37924
**Panel B: CARs for all legalizations, by brand status**						
	All years			No November 2016		
Day	Generic	Brand	Diff.	Generic	Brand	Diff.
-10	-0.0011	-0.0013**	0.0003	0.0036***	0.0023***	0.0013
-9	-0.0027***	-0.0019***	-0.0008	0.0017	0.0014**	0.0003
-8	-0.0035***	-0.0028***	-0.0007	0.0007	0.0008	-0.0001
-7	-0.0044***	-0.0057***	0.0013	0.0003	-0.0014*	0.0017
-6	-0.0079***	-0.0063***	-0.0017	-0.0042***	-0.0019**	-0.0023
-5	-0.0100***	-0.0066***	-0.0034**	-0.0080***	-0.0025***	-0.0055***
-4	-0.0096***	-0.0082***	-0.0014	-0.0068***	-0.0044***	-0.0024
-3	-0.0101***	-0.0079***	-0.0022	-0.0066***	-0.0043***	-0.0023
-2	-0.0080***	-0.0084***	0.0003	-0.0052***	-0.0051***	-0.0001
-1	-0.0120***	-0.0072***	-0.0048**	-0.0098***	-0.0041***	-0.0056***
0	-0.0132***	-0.0070***	-0.0062***	-0.0139***	-0.0065***	-0.0074***
1	-0.0100***	-0.0029***	-0.0071***	-0.0162***	-0.0057***	-0.0105***
2	-0.0053***	-0.0021*	-0.0033*	-0.0140***	-0.0060***	-0.0080***
3	-0.0024*	0.0013	-0.0037*	-0.0123***	-0.0029**	-0.0095***
4	-0.0021	0.0025**	-0.0046*	-0.0130***	-0.0017	-0.0113***
5	-0.0043***	0.0029***	-0.0073***	-0.0146***	-0.0005	-0.0141***
6	-0.0071***	0.0011	-0.0082***	-0.0174***	-0.0017	-0.0158***
7	-0.0102***	0.0002	-0.0104***	-0.0215***	-0.0021*	-0.0193***
8	-0.0096***	-0.0004	-0.0093***	-0.0206***	-0.0023*	-0.0183***
9	-0.0117***	-0.0001	-0.0116***	-0.0223***	-0.0017	-0.0207***
10	-0.0139***	-0.0001	-0.0138***	-0.0255***	-0.0011	-0.0243***
N	42516	27305	15211	24025	13899	37924

Notes: We show the combined effect of medical and recreational use legalizations. CARs are calculated using the Fama-French-Carhart model with a 100-day estimation window and a 50-day gap. We calculate ARs and CARs of a buy-and-hold strategy starting 20 trading days before the event. We show mean cross-sectional ARs and CARs (in %) in a 10-day window around the event.

* indicates significance at 10% level,

** at 5% level, and

*** at 1% level.

### Implied change in drugmaker sales

Table 6 presents estimated changes in annual total sales per event. We find the average change in a firm’s market value per legalization event is $63M with a total impact on market value across firms per event of $9.8B. Using the historical price-to-sales ratio of drugmakers for the year associated with each legalization event, this implies a change in annual sales across all drugmakers of $3B per event. When we separately assess changes for medical and recreational events, medical legalization implies an annual sales decrease of $2.4B. The implied sales decrease from recreational legalization is about 129% greater than that of medical legalization. Comparing effects on generic and brand drugmakers, we find the effect on brand drugmakers is 224% larger than the effect on generic drug maker sales.

**Table 6:** Estimated change in total annual sales in response to cannabis legalization.

	Mean	Median	SD
**Panel A: All events**			
Market value impact per firm x event	(63)	(2)	1,881
Market value impact per event	(9,794)	(14,546)	33,394
Total annual sales impact per event	(2,999)	(3,598)	7,835
**Panel B: Total Annual Sales Impact by Event Type**			
Medical legalization	(2,357)	(2,072)	8,180
Recreational legalization	(5,461)	(6,937)	6,833
Generic drugmakers	(147)	(191)	1,210
Branded drugmakers	(1,602)	(4,973)	9,392

Notes: This table presents the short-term economic impact of legalization events on the market valuation in 2019 $MM. In Panel A, in the first row we show summary statistics of the market value impact for a single firm. Note that some medical and recreational legalization events occur at the same time in different states.

## Discussion

Our results show that cannabis legalization is associated with a decrease in the stock market returns for pharmaceutical firms. Medical legalization generates a more muted effect on cumulative returns than recreational legalization but is more persistent. Generic firms are affected more in percentage terms, while brand firms are more affected in terms of magnitude due to their larger market value. The negative impact of cannabis legalization on pharmaceutical firm returns consistently occurs within twenty days prior to enactment, with the uncertainty resolving sooner for medical legalizations than for recreational legalizations.

The documented anticipatory behavior and the persistence of the effect follow the literature in finance and political science. The market anticipates the event with significant accuracy within the two weeks prior to the event, as has been documented in studies of prediction markets [32], stock market election responses [31] and stock market trading patterns in the days before announcements by the U.S. Federal Reserve [40], and the effect exhibits some reversion [33–35]. As predicted, recreational legalizations, which occur primarily through ballot initiatives, resolve uncertainty closer to the event than typically more predictable signings of legislation by state governors, with state governors having between 3 and 45 days to sign or veto legislation [41]. Following [35], we find that events that likely received less media attention are more persistent, i.e., effects in response to medical legalizations, which only affect a sub-population in a state, and for generic drugmakers, which are substantially smaller in market value than brand manufacturers.

### Competitive pressure from cannabis entry

Our finding that cannabis entry decreases returns for both generic and brand drug makers is novel. Generic entry tends to increase brand drug prices in what is known as the *generic entry paradox* [42]. We see four main reasons for why cannabis entry differs from conventional generic entry. First, patient reports and studies of prescription drug use following cannabis legalization indicate cannabis effectively treats several conditions simultaneously, unlike most conventional drugs, which are typically treatments targeted at and FDA-approved for a limited number of conditions. If cannabis use can address several medical conditions at once, the net effect on drug spending may be much larger than typical generic drug entry, which competes only with the original brand formulary and FDA-approved generic therapeutic equivalents. Second, generic entrants tend to have small advertising budgets [42], while cannabis advertising after legalization is widespread [43]. Third, barriers to entry for cannabis production are typically low, in stark contrast to entry costs for conventional generic drugmaking, meaning that, although cannabis production is smaller scale and state-specific, more cannabis producers are likely to enter simultaneously. In contrast, conventional generic manufacturers have large centralized operations and distribution networks across state and country borders, with their products each individually approved by the FDA. Lastly, in the case of recreational legalization, cannabis enters essentially as an over-the-counter medication or herbal supplement with lower barriers to access than conventional prescription drugs.

### Implied change in sales and conventional drug spending

The substantial documented changes in drug company sales from cannabis legalization imply investors expect a correspondingly large change in spending on conventional prescription and over-the-counter pharmaceuticals by consumers and insurers. (We do not include non-retail drug spending, e.g., inpatient drug spending, for which cannabis is not a likely alternative.) Therefore, we can use our estimates of the anticipated change in drug sales in response to state-level legalization to estimate the change in conventional pharmaceutical drug spending if those states that do not have legal cannabis were to legalize it. In 2017, prescription drug spending in the U.S. was estimated to be $333B [44], while an estimate from 2010 indicates an OTC market of about $23B [45]. Using the average medical legalization effect of $2.4B, if the remaining 16 states were to legalize medical cannabis alone, spending on conventional retail pharmaceuticals in the United States would decrease by $38.4B, or by about 10.8%. This estimate comes from the product of 16 states and the total annual sales impact per medical event of 2.4B in Table 6. For the percent change, we then divide by 333B plus 23B.

Our estimate of the impact of cannabis legalization on drug spending does not directly relate to previous studies. Even among other studies considering national-level spending, our estimates differ because we measure the total annual sales impact rather than payer-specific annual spending and capture a broader range of drugs. For comparison, [4] estimated fee-for-service Medicaid spending on prescription drugs following medical cannabis legalization. Using spending on treating nine conditions for which they found medical cannabis to be a likely substitute, [4] predicted annual prescription drug spending would have been $1B lower in 2014 if all 30 states without legal medical cannabis in 2014 had legalized medical cannabis. In addition to capturing a larger number of drugs, a larger number of conditions, and all payers, our estimate may be larger also because, unlike [4], who take drug prices as given, our estimate captures the competitive pressure on prices that cannabis puts on *both* brand and generic drugmakers for both prescription and over-the-counter drugs.

## Conclusion

Using a data set and estimation approach novel to health policy, we find evidence that investors predict legal cannabis access will significantly decrease sales of conventional pharmaceutical drugs. Legal cannabis applies competitive pressure to both generic and brand drug markets, across both classes of drugmakers. This makes legal cannabis distinct from typical brand drug patent expiration and generic drug entry [46–49] where typically only one drug, the drug coming off patent, and its substitutes are affected. Furthermore, cannabis can be purchased without a prescription and home cultivated, unlike any other conventional medication.

The economic significance of an estimated $9.8B loss in market value across firms per cannabis legalization event is extremely large, however our results should be interpreted cautiously. A key limitation is that we model investors as rational, which may overstate the economic significance of our results. Second, we are limited to publicly traded firms and past legalization events. Third, we note that estimates may be sensitive to our choice of using 150 to 50 days before the legalization event. Finally, we expect there to be measurement error due to heterogeneity in the legalization and subsequent regulatory processes. While measurement error would bias us towards finding no effect, the effects of assuming rational investor behavior could lead to an over- or under-estimate of the effect.

With these limitations in mind, the size of our estimated effect suggests important implications for stakeholders including drugmakers, patients and their providers, investors, regulators, and the academic community from both past and future legalization events. We discuss implications for each of these stakeholders below.

For private and public drugmakers, we expect the response to legalization to include investment and marketing. Recently, Pfizer paid $6.7B to acquire a biotech company that focuses on cannabinoid-type therapies [50]. Pharmaceutical firms have devoted substantial lobbying efforts and dollars into fighting cannabis legalization [51]. These are signs that the pharmaceutical industry from a marketing perspective, cannabis currently remains far from an FDA-approved therapeutic equivalent, and this might explain why pharmaceutical firms have spent less effort on detailing visits to doctors [52].

For patients, our results suggest that cannabis is a therapeutic alternative, however there are likely limits to substitution. Difficulties standardizing cannabis, limits on patentability, and a lack of knowledge about therapeutic mechanisms mean that cannabis’s potential to compete with FDA-approved medications is currently limited for many patients and their providers and may never fully compete for some. Given this, cannabis may operate as a drug outside the pharmaceutical marketplace for the foreseeable future, which may limit patient ability to pay for cannabis treatment through employer health insurance programs. Yet despite likely increased out-of-pocket expenses, our work and others’ suggests that many patients are substituting away from pharmaceutical drugs toward cannabis after legalization.

For investors, the negative impact of cannabis legalization on the market price indicates the adjustment of expectations about future cash flows of pharmaceutical firms. The size of the effect implies that participants in capital markets should monitor the evolving legal cannabis landscape as they diversify their portfolios. Given that we observe a market effect for each subsequent legalization, we expect that future events are not fully reflected in current equity prices. This suggests an opportunity for positive returns for any investment firm legally allowed to take short positions, which is equivalent to bets that the market valuation will decline in the future.

While current medical cannabis patients and their providers might not be surprised by the conclusions of this article, our results may help inform regulators of cannabis’ therapeutic potential. Our results suggest, given the breadth of cannabis’ therapeutic potential, that recreational users may even experience unexpected medical benefits. Population health implications will depend on how many people switch from conventional pharmaceutical medications to cannabis and the relative benefits, risks, and side effect profiles of the two treatments, as well as the costs and benefits of cannabis use for new cannabis consumers who were not previously consuming conventional medications.

Looking beyond effects for different stakeholder populations, our study suggests cannabis might be a useful tool for increasing competition in U.S. drug markets. Our use of stock return data permits us to estimate changes in conventional drug spending on drugs produced by publicly-listed firms for all payers and types of patients affected. We predict that if the remaining 16 states without medical cannabis legalization were to legalize cannabis, spending on conventional pharmaceutical drugs would decrease by almost 11%. We expect the introduction of a substitute to increase competition not just between the entrant and incumbent firms, but among incumbent firms as well as they jockey for market share. Moreover, pharmacy benefit managers, pharmacies, health insurers, and other members of the conventional pharmaceutical supply chain will experience spillover effects from more competition among drugmakers as it affects market power differentials throughout the supply chain.

The market’s recognition of cannabis as an alternative to conventional medications documented here underscores the need for additional research into the medical potential of cannabis, including phytochemicals beyond Tetrahydrocannabinol (THC) and Cannabidiol (CBD). The size of the response we see suggests that investors expect a large substitution away from conventional pharmaceuticals. A large shift toward therapeutic use of cannabis makes the need for research related to cannabis policy particularly urgent. Key areas include policies that regulate cannabis consumption to maximize benefits while minimizing costs urgent, e.g. consumption by minors, addiction, and impaired driving. Future research should also consider the effects of cannabis legalization on other players in the pharmaceutical supply chain and the returns on investment in cannabis markets for conventional pharmaceutical drugmakers.
